# MTP8 from *Triticum urartu* Is Primarily Responsible for Manganese Tolerance

**DOI:** 10.3390/ijms23105683

**Published:** 2022-05-19

**Authors:** Fanhong Wang, Kun Qiao, Huanhuan Wang, Hong Wang, Tuanyao Chai

**Affiliations:** 1 College of Life Sciences, Northwest Normal University, Lanzhou 730070, China; wangfanhong19@nwnu.edu.cn; 2 College of Life Sciences, University of Chinese Academy of Sciences, Beijing 100049, China; hhwang@ucas.ac.cn; 3 College of Horticulture and Landscape Architecture, Northeast Agricultural University, Harbin 150030, China; kunqiao@neau.edu.cn; 4 The Innovative Academy of Seed Design, Chinese Academy of Sciences, Beijing 100049, China

**Keywords:** CDF (cation diffusion facilitator), diploid wheat, transporter

## Abstract

Mineral nutrients, such as manganese (Mn) and iron (Fe), play essential roles in many biological processes in plants but their over-enrichment is harmful for the metabolism. Metal tolerance proteins (MTPs) are involved in cellular Mn and Fe homeostasis. However, the transporter responsible for the transport of Mn in wheat is unknown. In our study, TuMTP8, a Mn-CDF transporter from diploid wheat (*Triticum urartu*), was identified. Expression of *TuMTP8* in yeast strains of Δ*ccc1* and Δ*smf1* and *Arabidopsis* conferred tolerance to elevated Mn and Fe, but not to other metals (zinc, cobalt, copper, nickel, or cadmium). Compared with TuVIT1 (vacuole Fe transporter), TuMTP8 shows a significantly higher proportion in Mn transport and a smaller proportion in Fe transport. The transient analysis in tobacco epidermal cells revealed that TuMTP8 localizes to vacuolar membrane. The highest transcript levels of *TuMTP8* were in the sheath of the oldest leaf and the awn, suggesting that TuMTP8 sequesters excess Mn into the vacuole in these organs, away from more sensitive tissues. These findings indicate that *TuMTP8*, a tonoplast-localized Mn/Fe transporter, functions as a primary balancer to regulate Mn distribution in *T. urartu* under elevated Mn conditions and participates in the intracellular transport and storage of excess Mn as a detoxification mechanism, thereby conferring Mn tolerance.

## 1. Introduction

The micronutrient, manganese (Mn), is essential as a cofactor or an activator for many enzymes to regulate the catalysis of oxidation/reduction, decarboxylation and hydrolytic reactions [1]. Mn deficiency affects plant growth and development. However, excess Mn can also result in a reduction in growth, chlorophyll content and photosynthesis, damage to chloroplasts, and inhibition of enzyme activities [2,3]. Therefore, for normal growth, plants have developed various mechanisms to prevent over-accumulation and deficiency of Mn. Several proteins involved in Mn homeostasis have been characterized in *Arabidopsis* and rice, such as members of the cation exchanger (CAX), cation diffusion facilitator (CDF), P_2A_-type ATPase, natural resistance-associated macrophage protein (NRAMP), and ZRT/IRT-like protein (ZIP) families.

In *Arabidopsis thaliana*, both AtNRAMP1 and AtIRT1 are essential for Mn and Fe uptake [4]. *AtNRAMP1* is preferentially expressed in roots and its encoded protein localizes to the plasma membrane. Mutation of AtNRAMP1 was shown to reduce plant growth and Mn accumulation under insufficient Mn conditions, thus demonstrating that AtNRAMP1 is a high-affinity transporter for Mn uptake [5]. IRT1, a member of the ZIP family, is a high-affinity Fe transporter that was also confirmed as a transporter for Mn [6,7]. So, the mutated IRT1 has resulted in reduced Mn uptake by roots under Fe deficiency [7]. AtNRAMP3 and AtNRAMP4 play important roles in the export of vacuolar Mn [8], and AtMTP8, AtCAX2 and AtCAX4 sequestrate Mn into vacuoles to detract excessively absorbed Mn [9,10,11,12,13]. AtNRAMP2 is also a Mn transporter and localized in trans-Golgi network (TGN) and is required for optimal plant growth under Mn-deficient conditions [14]. Two proteins of the Ca-ATPase subfamily of the P-type ATPase superfamily, namely, AtECA1 and AtECA3, can also detoxify excess Mn by transporting Mn from the cytosol to the endoplasmic reticulum (ER) and Golgi [15]. AtMTP11 localizes to the Golgi/prevacuolar compartments and is required for Mn tolerance [16]. Two other members of the ZIP family, AtZIP1 and AtZIP2, participate in the translocation of Mn from roots to shoots [17].

In rice, the proteins OsNRAMP5 and OsMTP9 are involved in Mn uptake [18,19]. They show different polarities and function as an influx and efflux transporter, respectively, within the same cell at the exodermis and endodermis of mature root zones, where Casparian bands are localized [18,19]. At a low Mn concentration, the high expression of *OsNRAMP3* in the rice node plays a role in transporting Mn to young leaves and panicles. In contrast, under high-Mn concentrations, *OsNRAMP3* degraded rapidly to avoid Mn toxicity of young tissues [20]. The tonoplast-localized OsMTP8 and OsMTP8.1 also play important roles in the detoxification of excess Mn in rice [21,22].

The CDF proteins function as proton antiporters and are responsible for effluxing divalent cations from the cytoplasm to the outside of the cell or into subcellular compartments. Its members are involved in the transport of multiple divalent cations including zinc, Fe, cobalt, cadmium, and Mn [23,24]. As such, these transporters have been implicated in conferring metal tolerance and are designated as metal tolerance proteins (MTPs) in plants. Plant CDFs are phylogenetically clustered into three groups—Zn-CDF, Fe/Zn-CDF, and Mn-CDF—based on their putative specificity for transported metal ions [25].

As mentioned above, a number of transporters involved in the uptake of Mn from soils, its translocation to the shoot, and its storage in cells have been characterized in *Arabidopsis* and rice. However, there are few reports on the protein(s) that transport Mn in wheat. Diploid *Triticum urartu* is the progenitor of the A subgenome of hexaploid wheat (*T. aestivum*, AABBDD) [26]. Here, we identified and characterized metal tolerance protein 8 (MTP8), a member of the Mn-CDF group in *T. urartu*. We explored its role in Mn homeostasis by determining the expression profile of its encoding gene, the subcellular localization of its encoding protein, and the effects of its heterologous expression in yeast and *Arabidopsis*.

In this study, we found that *TuMTP8* was highly expressed in the third leaf-sheath and awn, and the encoded protein was localized to the tonoplast. We demonstrated that expression of TuMTP8 in yeast strains of Δ*ccc1* and Δ*smf1* conferred tolerance to an elevated level of Mn and, to a lesser extent, Fe, but not to other metals (Zn, Co, Cu, Ni, or Cd). Overexpression of *TuMTP8* in *Arabidopsis* conferred Mn tolerance and enhanced its growth relative to that of wild-type *Arabidopsis*. We concluded that TuMTP8 participates in detoxification by sequestering excess Mn into the vacuoles of *T. urartu* cells, thereby preventing damage to cellular processes and young tissues.

## 2. Materials and Methods

### 2.1. Plant Material and Growth Conditions

**Figure 1:**
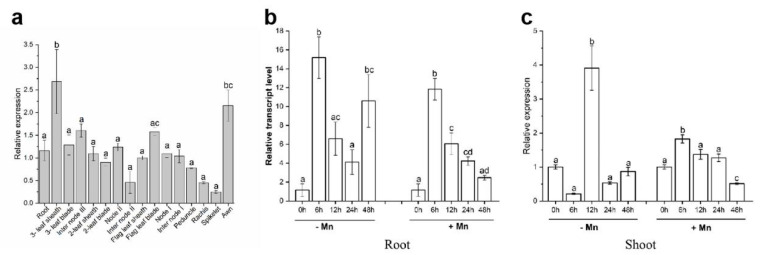
Tissue-specific expression patterns and expression profile of *TuMTP8* under Mn exposure or Mn deficiency. (**a**) The transcript levels of *TuMTP8* in 16 tissues were performed using qRT-PCR, and the gained data were calculated using the 2^−∆∆CT^ method with Ta2291 as the internal reference gene. n = 3. Significant differences are indicated by different letters (a, b, c, d). (**b**,**c**) The *T. urartu* seedlings were treated in ½ MS medium without or with 1 mM MnSO_4_ for 6 h, 12 h, 24 h, or 48 h, 0 h as the control. The shoot (**b**) and root (**c**) were collected, respectively, and the expression levels of *TuMTP8* in those parts were gained using the same methods as (**a**).

*T. urartu* (accession G1812) was grown hydroponically in half-strength Murashige and Skoog (½ MS) solution (pH 5.8). For the Mn treatment, 3-week-old plants were transferred into nutrient solution (pH 5.8) containing 1 mM MnSO_4_ for 6 h, 12 h, 24 h, and 48 h (0 h as control). To impose Mn deficiency, 3-week-old plants were transferred to ½ MS solution (pH 5.8) without MnSO_4_ for 6 h, 12 h, 24 h, and 48 h (0 h as control). For gene expression analyses, 16 organs of *T. urartu* plants (as shown in Figure 1a) were collected separately and frozen immediately in liquid nitrogen in the field. *Arabidopsis* plants (Columbia ecotype) were grown in an incubator under a 16h/8h photoperiod at 22 °C (day)/20 °C (night) and 125 μmol/m^2^/s photosynthetic photon flux density. After stratification at 4 °C for 2–4 days, seeds were sown in soil (to obtain and propagate plants transformed with *TuMTP8*) or in/on a ½ MS liquid medium or solid medium supplemented with 10 g/L agar. For the growth tests, the ½ MS solid medium was supplemented with or without 1 mM MnSO_4_, 180 μM FeSO_4_, 0.4 mM ZnSO_4_, 0.1 mM CoCl_2_, 0.02 mM CdCl_2_, 0.1 mM CuSO_4_, or 0.1 mM NiCl_2_. Tobacco (*Nicotiana tabacum*) plants were cultivated at 22 °C under a 16 h light/8 h dark cycle.

### 2.2. RNA Isolation, Reverse Transcription, and qRT-PCR

Total RNA was extracted from roots and shoots of *T. urartu* seedlings or 16 organs of *T. urartu* at the filling stage using the Plant Total RNA Purification Kit (GeneMarkbio, Taiwan, China). Reverse transcription was performed with 1–7 μL of total RNA using a PrimeScrit^TM^ RT reagent kit (TaKaRa Biotech, Otsu, Japan). Quantitative RT-PCR (qRT-PCR) was performed using 2× SYBR qPCR Master Mix (YEASEN Bio Inc., Shanghai, China) in a 96-well plate using a CFX 96 Touch Real-time PCR system (Bio-Rad, Hercules, CA, USA). The PCR mixture had a total volume of 10 μL, consisting of 1 μL of diluted cDNA template, 0.4 μL each primer (10 μM), 3.2 μL nuclease-free water, and 5 μL 2× SYBR qPCR Master Mix. The primers qTuMTP8-F and qTuMTP8-R were used to amplify part of the *TuMTP8* gene and the primers Ta2291_F/Ta2291_R were used to amplify the internal reference genes (Table S1) [27]. The reaction conditions for qRT-PCR were 95 °C for 5 min followed by 40 cycles of 95 °C for 5 s, and 60 °C for 30 s. Each reaction was repeated three times. Relative gene expression levels were calculated using the 2^−△△CT^ method and the mean ± SE for all replicates was calculated for each data point.

### 2.3. Expression of TuMTP8 in Yeast

The yeast (*Saccharomyces cerevisiae*) wild-type strain BY4741 (MATα; *his3Δ1*; *leu2Δ0*; *met15Δ0*; *ura3Δ0*), the Mn-sensitive Δ*smf1* strain (BY4741; MATα; *his3Δ1*; *leu2Δ0*; *met15Δ0*; *ura3Δ0*; YOL122c:: kanMX4), and the Mn- and Fe-sensitive Δ*ccc1* strain (BY4741; MATα; *his3Δ1*; *leu2Δ0*; *met15Δ0*; *ura3Δ0*; YLR220w::kanMX4) were used in this study.

The coding sequence of *TuMTP8* was amplified from *T. urartu* cDNA using Phanta Max Super-Fidelity DNA Polymerase (Vazyme Biotech, Nanjing, China) with the primers TuMTP8-F and TuMTP8-R (Table S1). The amplified product was purified by 1.0% agarose gel electrophoresis and then subcloned into the pCloneEZ-Blunt-Amp/HC cloning vector (CloneSmarter, Houston, TX, USA). The pYES2-TuMTP8 vector was produced by amplifying *TuMTP8* with the primers (Table S1); and then introducing the fragment into the *Hind* Ⅲ and *Eco*R Ⅰ sites of pYES2 (Invitrogen, Carlsbad, CA, USA) by homologous recombination (ClonExpress^TM^ One-Step Cloning Kit, Vazyme Biotech, China). The sequences were confirmed by sequencing. pYES2-TuMTP8 or the pYES2 vector alone was introduced into yeast strains by the LiOAc/polyethylene glycol (PEG) method [28]. Yeast transformants were selected on synthetic defined (SD) medium lacking uracil (SD-Ura, pH 5.8) containing 2% (*w/v*) glucose (Glu) as the carbon source; meanwhile, the empty vector pYES2 and pYES2-TuMTP8 with the *GAL1* promoter were expressed in yeasts on SD-Ura medium containing 2% (*w/v*) galactose (Gal) as the carbon source.

### 2.4. Yeast Growth Assay

Yeast strains transformed with pYES2-TuMTP8 or pYES2 were cultured in liquid SD-Ura/Glu medium (pH 5.8) up to the exponential phase at 30 °C overnight. Yeast cultures were harvested by centrifuging, washed, and resuspended to OD_600nm_ = 0.5 in sterile ddH_2_O. This suspension was used to produce four 10-fold serial dilutions in the same sterile ddH_2_O for each culture. Then, 5 μL of each dilution was spotted onto solid YP plates with Gal or Glu and different concentrations of FeCl_3_, ZnSO_4_, FeSO_4_, CdSO_4_, NiCl_2_, CoCl_2_, MnSO_4_, or CuSO_4_. YPD plates were used as a control drop assay. After incubating at 30 °C for 3 to 5 days, plates were photographed.

For the liquid growth assay, the yeast transformants of Δ*ccc1* were diluted in 20 mL liquid SD-Ura medium supplemented with 2% Gal to an initial OD_600nm_ = 0.1 in a 50 mL centrifuge tube with or without 10 mM MnSO_4_ or 5 mM FeSO_4_. Then, the tubes were shaken at 30 °C at 200 rpm, and the OD_600nm_ was measured in 0.1 mL samples collected after 4 h, 8 h, 12 h, 24 h, 36 h, and 48 h, respectively.

### 2.5. Metal Transport in Yeast Cells

For the Mn accumulation test, the yeast transformants of BY4741 were shaken at 30 °C for 24 h in liquid SD-Ura medium with Gal. The cultures were diluted to OD_600nm_ = 0.6 in 20 mL medium containing 3 mM MnSO_4_ and incubated at 30 °C for 48 h. The yeast samples were collected after centrifugation and washed three times using 10 mM Na_2_EDTA and deionized H_2_O, respectively. The samples for Mn measurement were dried at 50 °C for 48 h, weighed, and then treated using a mixture of HNO_3_ and H_2_O_2_ in an 8:3 volume ratio at 150 °C for 1 h. The digested fluid was fixed volume to 25 mL with deionized H_2_O. The Mn concentrations in each sample were measured using inductively coupled plasma optical emission spectrometry (ICP-OES, Perkin Elmer, Waltham, MA, USA). The experiment was performed three times.

### 2.6. Transient Expression of TuMTP8 in Tobacco

A fragment of *TuMTP8* lacking the stop codon and with an added two nucleotides guanine (GG) was amplified with the primers TuMTP8_pEGFP&pBI121_F(B) and TuMTP8_pEGFP&pBI121_R(K) (Table S1) and cloned into the *Bam*H I and *Kpn* I sites of pBI121-eGFP (harboring the gene encoding enhanced green fluorescent protein) using a homologous recombination system to construct pBI121-TuMTP8-eGFP.

The construct was introduced into epidermal cells of 4-week-old tobacco plants for transient expression of the TuMTP8-eGFP fusion proteins. The pBI121-TuMTP8-eGFP construct was first introduced into *Agrobacterium tumefaciens* (strain GV3101) as described elsewhere [29]. *A. tumefaciens* transformed with TuMTP8-eGFP was cultured at 28 °C overnight (~16 h), and then further cultured in fresh medium containing 10 mM MES and 20 μM acetosyringone (AS) with shaking at 28 °C overnight. After centrifugation, the cell pellet was resuspended in infiltration buffer (MES 10 mM, pH 5.6, AS 150 μM (diluted from a 100 mM stock in dimethyl sulfoxide), MgCl_2_ 10 mM) to OD_600nm_ = 1.5. Cells containing P19, an inhibitor of gene silencing, was cultured in the same way to OD_600nm_ = 1. The two bacterial suspensions were mixed at equal volumes. The mixture was introduced using a 1 mL needleless syringe by gentle pressure through the stomata on the lower epidermal surface of the leaf [30]. Transformed plants were kept in the dark for 1 day, then grown in the light for 2 days at 22 °C. The eGFP fluorescence in transformed leaves was observed and photographed under a fluorescence microscope.

### 2.7. Plant Transformation

The *TuMTP8* coding sequence was inserted into the *Bam*H I and *Sac* I sites of the pBI121 binary vector, which harbors the 35S promoter and confers kanamycin resistance in transgenic plants. pBI121 containing *TuMTP8* was introduced into *A. tumefaciens* through heat shock after liquid-nitrogen cryogenic treatment. *Arabidopsis* plants with many buds were subsequently transformed by *A. tumefaciens*-mediated transformation using the floral-dip method [31]. Seeds from the first generation of transformed plants were selected on ½ MS medium (pH 5.8) containing 0.8% (*w/v*) agar and 35 mg/L kanamycin (Kan). These transformed plants were further confirmed by sequencing. In total, 7–15 T_1_ transgenic seedlings were obtained for each transformation and were used for selecting T_2_ transgenic plants resistant to Kan.

### 2.8. Stress Tolerance of Arabidopsis Overexpressing TuMTP8

To determine the effect of TuMTP8 on metal tolerance, T_2_ transgenic *Arabidopsis* seeds were germinated on ½ MS solid plates supplemented with or without 1 mM MnSO_4_, 180 μM FeSO_4_, 0.4 mM ZnSO_4_, 0.1 mM CoCl_2_, 0.02 mM CdCl_2_, 0.1 mM CuSO_4_, or 0.1 mM NiCl_2_. The seedlings were grown for 14–16 days. Photographs were taken using a NIKON 5200 digital camera (Nikon, Tokyo, Japan) and the root length and the fresh weight were determined.

### 2.9. Statistical Analysis

Data were analyzed using Student’s *t* test (Excel) and Tukey’s test, or Dunnett’s test (One-way ANOVA; SPSS 20). OriginPro 2017 was used to fit the data. In figures, significant differences are indicated by asterisks (*, *p* < 0.05; **, *p* < 0.01) and different letters (a, b, c, d, or e), respectively.

## 3. Results

### 3.1. Molecular Features of TuMTP8

**Figure 2:**
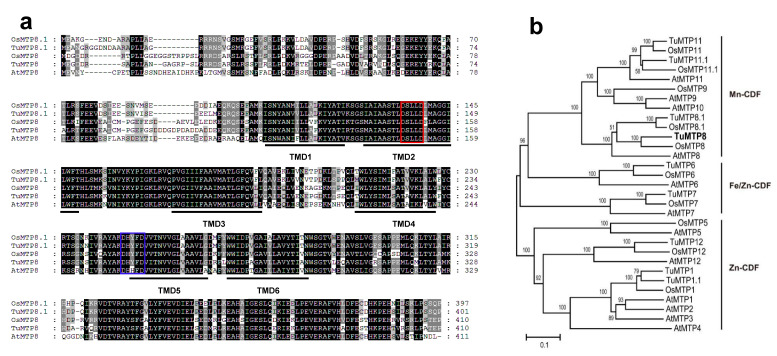
Sequence alignment and phylogenetic relationship analysis of MTP8-like proteins from *T. urartu*, *A. thaliana*, and *O. sativa.* (**a**) The alignment was performed using ClustalW, and visualized using GeneDoc. MTP8-like proteins contain TuMTP8, TuMTP8.1, OsMTP8, OsMTP8.1, and AtMTP8. Identical or similar amino acid residues were represented by black and gray shade, respectively. Red and blue boxes indicate the conserved sequences DSLLDD or DHYFD in TMDs II and V, respectively. (**b**) The neighbor-joining tree was built in MEGA 5.2 with the following parameters: pairwise deletion, p-distance, and 5000 replications. TuMTP8 from *T. urartu* were highlighted in bold. Accession numbers of all MTP members from *T. urartu*, *A. thaliana*, and *O. sativa* are listed in Table S2.

The full coding sequence of the putative *TuMTP8* gene was obtained by BLAT searches of the *T. urartu* genome using the amino acid sequences of AtMTP8 and OsMTP8 (At3g58060 and LOC_Os02g53490) as the search queries. The sequence was confirmed to be identical to that of the *TuMTP8* fragment amplified by PCR amplification from cDNA of *T. urartu* (accession no. MH464868). *TuMTP8* was mapped to chromosome 6 (TuG1812G0600003452). Alignment between the gene sequence and the full coding sequence revealed six introns. The TuMTP8 protein was deduced to contain 410 amino acid residues. Online bioinformatics tools revealed six transmembrane domains (TMDs) in TuMTP8 (Figure S1; SACS HMMTOP), a cytoplasmic N-terminus, and two conserved motifs: DSLLD in the putative TMDII, and DHYFD in the cytosolic loop preceding TMDV common for MTP8-like transporters (Figure 2a). In the phylogenetic tree constructed using *Arabidopsis* and rice MTP sequences, TuMTP8 showed the highest similarity to OsMTP8 (Figure 2b). The amino acid sequence of TuMTP8 was 80.19% and 61.35% identical to those of OsMTP8 and AtMTP8, respectively.

### 3.2. Expression Pattern of TuMTP8

To investigate the tissue-specific expression patterns of *TuMTP8* and its responses to Mn, the mRNA levels of *TuMTP8* in 16 organs of *T. urartu* at the filling stages were determined by qRT-PCR. The highest transcript levels of *TuMTP8* in *T. urartu* were obtained in the sheath of the third leaf (the oldest leaf) followed by the awn (Figure 1a).

Furthermore, the expression profiles of *TuMTP8* in both shoots and roots were compared in the presence or absence of Mn. Under exposure to 1 mM Mn, *TuMTP8* was up-regulated in roots and shoots at all time points except for 48 h. The transcript levels of *TuMTP8* initially increased and then gradually decreased in both roots and shoots over time. In roots, the transcript levels of *TuMTP8* first increased and then gradually decreased from 6 h to 12 h, 24 h, and 48 h, but were still higher at 48 h than at 0 h (Figure 1b,c). In shoots, the transcript level of *TuMTP8* at 48 h of Mn exposure was lower than that at 0 h (Figure 1b,c). Under Mn deficiency, the transcript levels of *TuMTP8* fluctuated in both roots and shoots: the highest transcript level of *TuMTP8* in roots was at 6 h, while that in shoots was at 12 h (Figure 1b,c). Compared with that in shoots, the transcript level of *TuMTP8* in roots was significantly increased by both excess Mn and Mn deficiency (Figure 1b,c). These results show that *TuMTP8* was induced by excess Mn and Mn deficiency, especially in the roots.

### 3.3. TuMTP8 Conferred Mn and Fe Tolerance in Yeast

**Figure 3:**
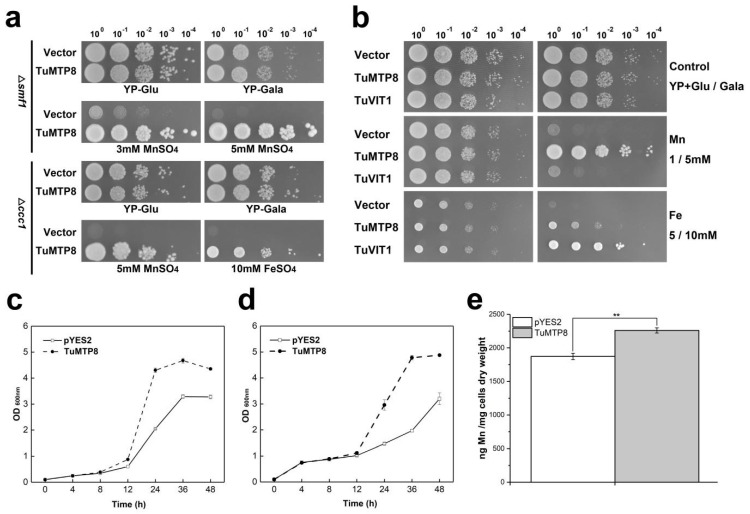
Functional assay of TuMTP8 in yeast strains. (**a**) The growth of the Δ*smf1* and Δ*ccc1* strain transformed with either the empty vector pYES2 or pYES2 carrying *TuMTP8* under 3 mM or 5 mM MnSO_4_ or 10 mM FeSO_4_ was compared. Four 10-fold serial dilutions were prepared from yeast cultures adjusted to an OD_600nm_ of 0.5. Each dilution (5 μL) was spotted on YP-Gal plates supplemented without or with different concentrations of FeSO_4_ or MnSO_4_ or onto YP-Glu plates (control). The plates were placed in the incubator at 30 °C for 2–5 days. (**b**) Comparative analysis of TuMTP8 and TuVIT1 expression in yeast *Δccc1* strains. The growth of Δ*ccc1* strain transformed with either the empty vector pYES2 or pYES2 carrying *TuMTP8* or *TuVIT1* under 1 or 5 mM MnSO_4_ or 5 or 10 mM FeSO_4_ was compared. Four 10-fold serial dilutions were prepared from yeast cultures adjusted to an OD_600nm_ of 0.5. Each dilution (5 μL) was spotted on YP-Gal plates supplemented without or with MnSO_4_ (1 mM or 5 mM) or FeSO_4_ (5 mM or 10 mM) or onto YP-Glu plates (control). The plates were cultured at 30 °C for 2–5 days. (**c**,**d**) Growth curve of yeast mutant *Δccc1* expressing TuMTP8 and containing empty vector under Mn or Fe exposure. Δ*ccc1* strain transformed with either the empty vector pYES2 or pYES2 carrying *TuMTP8* was cultured in the liquid SD-Ura (Glu) medium. After washing with aseptic ultra-pure water twice, the yeasts were diluted to an OD_600nm_ = 0.1 in 20 mL liquid SD-Ura (Gal) medium containing 10 mM MnSO_4_ or 5 mM FeSO_4_. The cultures were then shaken at 30 °C at 200 rpm. The OD_600nm_ values of the cultures in the presence of Mn (**c**) or Fe (**d**) were measured at 0 h, 4 h, 8 h, 12 h, 24 h, 36 h and 48 h. The growth of yeast transformed with the empty vector *pYES2* and expressing *TuMTP8*, were indicated by the open symbols and the filled symbols, respectively. n = 3. (**e**) Mn content for BY4741 carrying *TuMTP8* (gray bar) compared to empty vector (white bar). The initial OD_600nm_ of the cultures were diluted to 0.6 in 20 mL medium containing 3 mM MnSO4 and incubated at 30 °C for 48 h. Data are represented as means ± SE, n = 3. “**” (*p* < 0.01) represents significant difference between the *TuMTP8*-expressing yeast and the yeast containing empty vector.

To characterize the function of *TuMTP8*, we cloned its encoding gene into yeast strains and determined whether it affected their tolerance to metals. Cultures of the yeast wild-type strain BY4741 carrying either pYES2 or pYES2-TuMTP8 grew similarly in medium containing no metal ions or any of the tested ions apart from Mn and Fe (data not shown). Introduction of *TuMTP8* complemented the Mn-sensitive phenotype of the Δ*smf1* and Mn-/Fe-sensitive phenotype of the Δ*ccc1* on YP+Gal medium supplemented with 3/5 mM Mn or 10 mM Fe (Figure 3a). The growth of the Δ*ccc1* transformed with the empty vector was inhibited in liquid medium containing Mn or Fe, in contrast to Δ*ccc1* harboring the *TuMTP8* (Figure 3c,d). Next, the ability of TuMTP8 and TuVIT1 (vacuole iron transporter1 from *T. urartu*) to transport Fe and Mn was compared by monitoring the growth of Δ*ccc1* transformed with *TuVIT1* or *TuMTP8* in YP+Gal medium supplemented with 5/10 mM Fe or 1/5 mM Mn. Compared with Δ*ccc1* harboring *TuVIT1*, Δ*ccc1* harboring *TuMTP8* showed significantly higher Mn tolerance but lower Fe tolerance (Figure 3b). These results suggest that in *T. urartu*, TuMTP8 is primarily responsible for Mn tolerance, although it contributes to Fe transport to some extent. Next, we used ICP-OES to determine the Mn content in Δ*ccc1* carrying the empty vector or *TuMTP8* cultivated in 3 mM Mn medium for 48 h. Compared with the Δ*ccc1* carrying the empty vector, the Δ*ccc1* carrying *TuMTP8* accumulated significantly more Mn (Figure 3e, *p* < 0.01), suggesting that TuMTP8 can detoxify Mn in yeast cells by internal sequestration of Mn, rather than efflux of Mn to the external medium.

### 3.4. TuMTP8 Localized to the Vacuolar Membrane of Plant Cells

**Figure 4:**
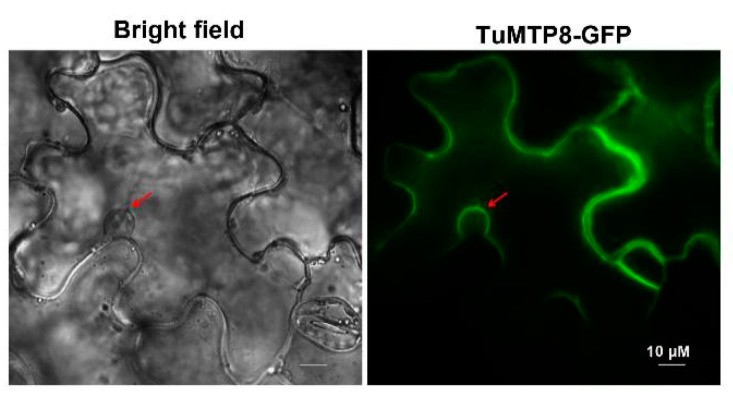
Subcellular localization of TuMTP8 in a tobacco epidermal leaf cell. *Agrobacterium* transformed with *TuMTP8-eGFP* was injected in tobacco. The tobacco was cultured in the dark for one day and in light for two days, and then was screened. The figure exhibits the bright-field (**Left panel**) and eGFP fluorescence (**Right panel**). The arrow indicated cell nucleus. Scale bar = 10 μm.

To observe the subcellular localization of *TuMTP8*, a fusion protein was generated by introducing the *TuMTP8* sequence into the pBI121 vector with eGFP at the C-terminus. Transient expression in tobacco epidermal cells followed by fluorescence microscopy showed that the GFP signal in the leaves expressing TuMTP8-eGFP was observed overlapped with plasma membrane of a cell except for isolating the cell nucleus, wihch is characteristic of the vacuolar membrane of central large vacuoles (Figure 4). This result indicated that TuMTP8 localize to the tonoplast of plant cells. This was supported by the fact that TuMTP8 enhanced Mn tolerance and elevated Mn accumulation in yeast cells (Figure 3a,c,e).

### 3.5. Expression of TuMTP8 in Arabidopsis Conferred Mn Tolerance

**Figure 5:**
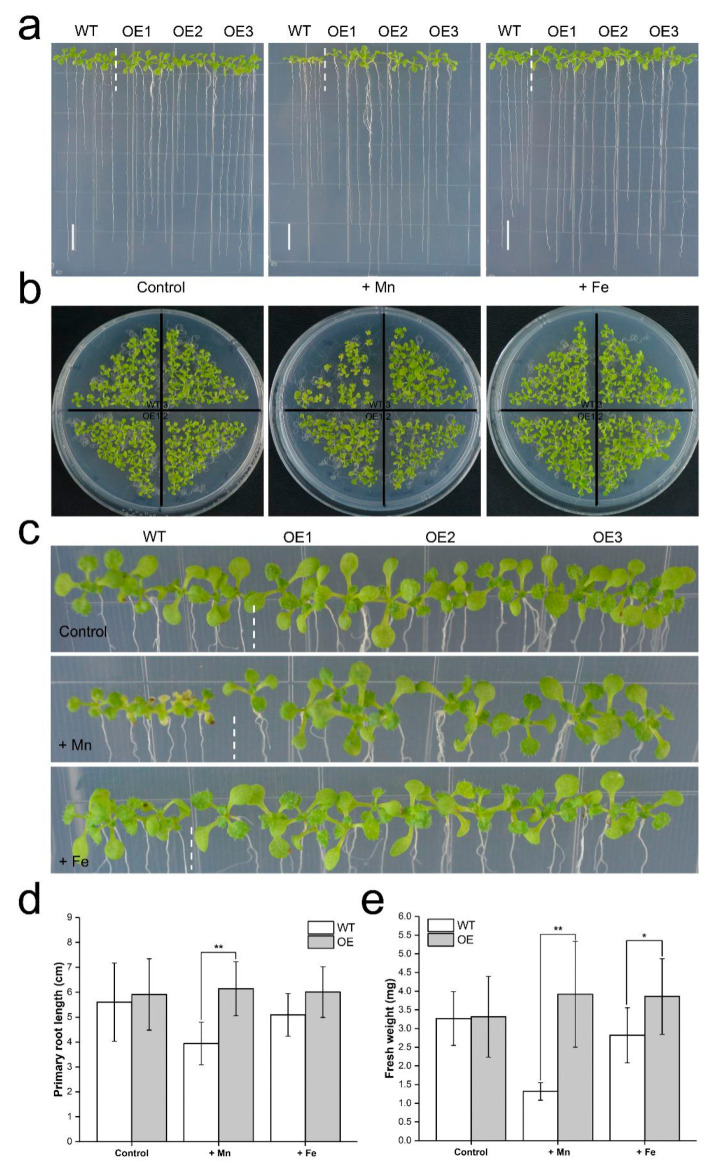
Effects of MnSO_4_ and FeSO_4_ stress on growth of *Arabidopsis* over-expressing *TuMTP8* compared to WT plants. (**a**) WT and transgenic *Arabidopsis* seeds were grown on vertical ½ MS solid medium supplemented without (Control) or with 1 mM MnSO_4_ and 100 μM FeSO_4_, respectively. Seedlings were grown in an incubator under a 16 h/8 h photoperiod at 22 °C (day)/20 °C (night) for 14 days. Bars = 1 cm. (**b**) WT and transgenic seeds were grown on ½ MS solid medium supplemented without (Control) or with 1 mM MnSO_4_ and 100 μM FeSO_4_, respectively. Seedlings were grown for 30 days. (**c**) Enlarged images of the shoots in (**a**). (**d**,**e**) Root length (**d**) and fresh weight (**e**) of WT and *TuMTP8*-OE plants described in (**a**). Three biological repeats were performed with similar results. Four to six plantlets per genotype from one plate were measured for each repeat. Data are presented as means ± SE, n ≥ 5. Bars with asterisk (*) are different at *p* < 0.05, and with (**) are significantly different at *p* < 0.01.

To determine whether enhanced expression of *TuMTP8* was responsible for the enhanced Mn accumulation in the vacuoles leading to the increased Mn tolerance in plants, we generated transgenic *Arabidopsis* plants over-expressing *TuMTP8*. To test the effect of excess Mn on plant growth, three independent homozygous lines (OE-1, OE-2, and OE-3) were used, with wild-type *Arabidopsis* (WT) as the control. When grown on ½ MS medium, the three transgenic lines were not noticeably different from WT (Figure 5a–c). In contrast, under 1 mM MnSO_4_ treatment, root growth was significantly enhanced in *TuMTP8*-overexpressing *Arabidopsis* lines relative to WT (Figure 5a,d; *p* < 0.01). In addition, the shoots of the over-expressing lines grew well, whereas those of WT showed obvious chlorosis and impaired growth (Figure 5a–c). The fresh weight of the entire seedling was greater in the over-expressing lines than in the WT under 1 mM MnSO_4_ treatment (Figure 5e; *p* < 0.01) and 180 μM Fe treatment (Figure 5e; *p* < 0.05). No differences in growth were observed between the over-expressing lines and WT when the plants were grown with other metals (data not shown). Upon exposure to Mn, over-expressing lines accumulated less reactive oxygen species (ROS) than WT, as determined by both DAB and NBT staining (Figure S2). Thus, the over-expressing lines showed enhanced tolerance to excess Mn.

**Figure 6:**
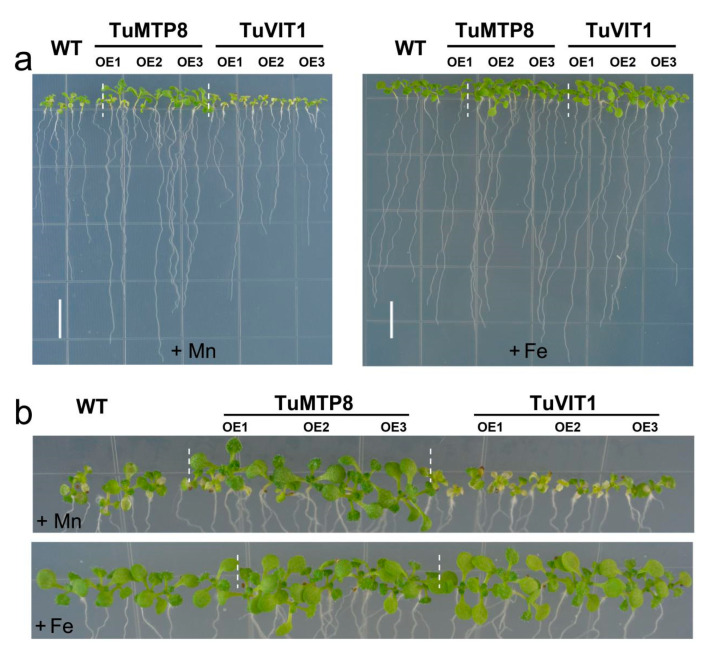
Comparison of growth between over-expressing *TuMTP8* and over-expressing *TuVIT1 Arabidopsis*. (**a**) WT, *TuMTP8*-OE and *TuVIT1*-OE transgenic *Arabidopsis* seeds were grown on vertical ½ MS solid medium supplemented with 1 mM MnSO_4_ and 100 μM FeSO_4_, respectively. Seedlings were grown for 14 days. Bars = 1 cm. (**b**) Enlarged images of the shoots in (**a**). Three biological repeats were carried out with similar results. Four to six plantlets per genotype from one plate were measured for each repeat.

To further explore the effects of TuMTP8 on Mn and Fe tolerance in plants, we compared tolerance to Mn and Fe between *TuMTP8*-overexpressing *Arabidopsis* lines and *TuVIT1*-overexpressing *Arabidopsis* lines. The results show that TuMTP8 had a limited effect on Fe tolerance but conferred significant Mn tolerance, whereas TuVIT1 resulted in Mn sensitivity and affected Fe tolerance (Figure 6). These findings further confirmed that TuVIT1 is a minor player in Mn tolerance as against *TuMTP8*.

## 4. Discussion

### 4.1. Tonoplast-Localized TuMTP8 Was a Mn and Fe-Specific Transporter

Although Mn is essential for plant growth and development, it can be toxic to plants in excess. In this study, we identified a Mn-CDF transporter, MTP8 from *T. urartu*, with six TMDs and two DxxxD motifs in TMDⅡ and in the cytosolic loop preceding TMDV, respectively. Heterologous expression of *TuMTP8* in yeast resulted in enhanced Mn tolerance and accumulation and, to a lesser extent, tolerance to Fe (Figure 3a,b). However, it did not confer tolerance to other heavy metals (data not shown). Similarly, besides Mn, AtMTP8 is also able to transport Fe [32]. However, some MTP8-like proteins that have been characterized in other plant species are highly specific to Mn. For example, OsMTP8 and OsMTP8.1 from rice and CsMTP8 from cucumber are specific Mn transporters [21,22,33]. In contrast, Mn transporters with broad substrate ranges are widely found in plants. For example, the multi-substrate transporter IRT can transport Fe, Mn, Co, Cd, and Zn [6,7], and NRAMP1 can transport Mn, Fe, and Co [4,5].

Heterologous expression of *TuMTP8* in *Arabidopsis* shows that the *TuMTP8-*overexpressing lines were able to grow normally in medium containing Mn at high levels, whereas the WT seedlings showed reduced growth and chlorosis (Figure 5). In addition, *Arabidopsis* expressing *TuMTP8* accumulated less ROS and thus high antioxidative enzymes activity than WT (Figure S2). No differences in growth between *Arabidopsis* expressing *TuMTP8* and WT were observed when plants were grown with other metals in the medium. Compared with WT and the lines expressing *TuVIT1*, the lines expressing *TuMTP8* were more tolerant to excess Mn providing further evidence that TuMTP8 is primarily responsible for Mn tolerance (Figure 6). Our subcellular localization analyses revealed that TuMTP8 is located on the vacuolar membrane (Figure 4), like many MTP8-like proteins in other plants (ShMTP8, OsMTP8, OsMTP8.1, AtMTP8, and CsMTP8). To date, most characterized MTP8-like proteins have been found to be located on the vacuole membrane [21,22,32,33,34]. Some exceptions are HvMTP8.1 and HvMTP8.2 from barley and MTP8 from *Camellia sinensis*, which localize to the Golgi and plasma membrane, respectively [35,36]. The results of our study and other studies suggest that localization to the vacuolar membrane is conserved among MTP8-like proteins. TuMTP8 localized to the vacuolar membrane and conferred yeasts to remarkable Mn tolerance. As the Mn content increases in yeasts, cells are protected by sequestration of excess Mn into the vacuole.

### 4.2. Corresponding Solutions of TuMTP8 to Mn Toxicity

We analyzed the expression pattern of *TuMTP8,* and detected increased transcript levels of *TuMTP8* under Mn excess and Mn deficiency, especially in the roots (Figure 1a–c). In cucumber, *MTP8* was shown to be up-regulated under excess Mn and down-regulated under Mn deficiency [33]. In contrast, the transcript levels of *OsMTP8* and *OsMTP11* in rice shoots and roots were found to be unaffected by low- or high-Mn conditions [22,37]. *TuMTP8* transcripts were more abundant in the sheath of the oldest leaf (third leaf) and the awn than in other organs (Figure 1a). Other studies have also found that the awn is involved in drought tolerance [38]. Awn has been well-established as an important structure of involvement in the transport of Mn [39]. Therefore, the higher expression level of *TuMTP8* in the awn may be a relevant factor in increased Mn tolerance. We can speculate that the high level of *TuMTP8* expression in the awn may be indicative of a protective function, whereby Mn is sequestered into vacuoles of the cells in the awn, away from other sensitive organs. The highest *TuMTP8* transcript levels were in the sheath of the oldest leaf. This may represent a strategy to protect young leaves and reproductive organs of *T. urartu* from excessive Mn toxicity by TuMTP8-mediated Mn influx into vacuoles in the cells of old leaves. In rice, the transcript levels of *OsYSL6*, encoding a Mn-nicotianamine transporter, were found to increase with leaf age, and its encoded protein was shown to detoxify excess Mn by transporting it into older leaves for storage [40]. In the case of *OsNRAMP3,* it was found to be degraded rapidly under high-Mn conditions, resulting in translocation of Mn to older tissues [41]. Previous reports have shown that *OsMTP11* and *OsMTP8* are predominantly expressed in older leaf blades, which accumulated more Mn than other tissues [22,37]. It was suggested that under high-Mn concentrations, old leaves had more Mn than young leaves. A few studies have reported that the high Mn tolerance of old leaves highly depends on the activity of Mn-CDF family transporters [37]. The fact that *TuMTP8* transcripts were most abundant in the awn and old leaves suggests that TuMTP8 might preferentially transport Mn to these tissues, thereby protecting young leaves and other parts from excess Mn. Since TuMTP8 localizes at the vacuolar membrane in tobacco epidermis cells, we can speculate that the increased Mn accumulation and resistance phenotype of plants expressing *TuMTP8* results from the TuMTP8-mediated transport of Mn into the vacuoles of the cells in the older leaf sheath and awn.

### 4.3. Possible Selection Mechanism of TuMTP8 on Mn and Fe

Our results show that TuMTP8 also transports Fe in addition to Mn. Compared with TuVIT1, a Fe transporter, TuMTP8 confers enhanced Mn tolerance and slight Fe tolerance (Figure 2b and Figure 6). Therefore, TuMTP8 is primarily responsible for Mn homeostasis, whereas TuVIT1 determines Fe storage. These roles are similar to those of AtVIT1 and AtMTP8 in *Arabidopsis* [13,32,42]. Previous studies have shown that those two transporters can replace each other: AtVIT1 can substitute for AtMTP8 when the latter is non-functional and vice versa [13,32].

Interestingly, many Mn transporters also have an affinity for Fe. For example, VIT1 homologous yeast CCC1 (Ca-sensitive cross complementer 1) transports Fe and Mn into the vacuole [43]. AtNRAMP1 is a high-affinity transporter for Mn uptake and a component of the low-affinity Fe transport system [4,5]. AtIRT1 is a high-affinity Fe transporter that can also transport Mn. Castaings et al. demonstrated that AtNRAMP1 and AtIRT1 are cooperatively required for Mn and Fe uptake [4]. Thus, Fe and Mn may be transported by common transporters and compete for absorption [5]. If this is the case, then these transporters must have a mechanism to selectively transport these substrates. It may be that a transporter can bind to a different substrate when other substrates are lacking or excessive. It was reported that excess Fe can compete with Mn and trigger Mn deficiency [5]. Additionally, AtMTP8 is responsible for Fe storage when AtVIT1 is non-functional, and AtVIT1 is responsible for the storage of Mn when AtMTP8 is disrupted [13]. Another mechanism may be differences in the temporal-spatial expression of genes encoding transporters. For example, the tonoplast-localized AtNRAMP3 and AtNRAMP4 function in Fe mobilization in germinating seeds, but are involved in Mn homeostasis in adult plants [8,44]. Similarly, AtMTP8 is responsible for Mn homeostasis during seed development and for Fe reallocation during seed germination [32].

### 4.4. Comparative Analysis of TuMTP8 and TaMTP8

The three homologous genes of TuMTP8 by BLAST alignment to the *T. aestivum* genome were found. They were assigned as TaMTP8-A, TaMTP8-B, and TaMTP8-D based on the location of their subgenome. Like *TuMTP8*, all of these three genes possess six introns and seven exons and are located on Chr. 6. The sequence of TuMTP8 is the same as that of TaMTP8-A. TuMTP8 has 99.02% and 98.78% sequence identity with TaMTP8-B and TaMTP8-D, respectively (Figure S3). Evidently, the information of TuMTP8 provides a clue for obtaining three TaMTP8 from the huge and complex wheat genome, and the extremely high sequence similarity and conserved structure of TuMTP8 and TaMTP8 (A, B, and C) indicate that they have a great functional similarity in Mn transport. This will help us understand the molecular mechanisms on Mn tolerance of *T. aestivum* and provide a theoretical reference for molecular breeding of wheat.

## 5. Conclusions

In conclusion, our results show that TuMTP8 is a vacuole membrane-localized Mn influx transporter that deals with Mn toxicity through internal sequestration of Mn into vacuoles of cells in the awn and sheath of old leaves in diploid wheat. A similar function of MTP8 from *T. aestivum* could be speculated and provide an important resource for future wheat breeding in Mn homeostasis under conditions of excessive Mn. TuMTP8 transports Mn, and to a lesser extent, Fe. Further research is required to explore the selective mechanism of TuMTP8 for Mn or Fe and to identify the key amino acid residues for its function.

## Data Availability

Not applicable.

## Supplementary Materials

The following supporting information can be downloaded at: https://www.mdpi.com/article/10.3390/ijms23105683/s1.
