# Supplementary figures and images for: MTP8 from Triticum urartu Is Primarily Responsible for Manganese Tolerance
Source: Int J Mol Sci. 2022 May 19;23(10):5683. doi: 10.3390/ijms23105683 (PMC9144917; doi:10.3390/ijms23105683)

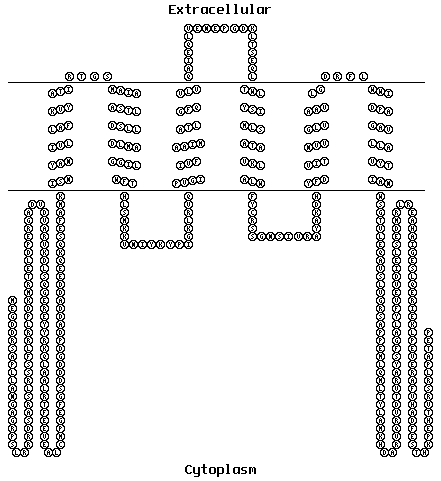

Supplement: Supplementary file 1 [file ijms-23-05683-s001.zip › Fig.S1.png]

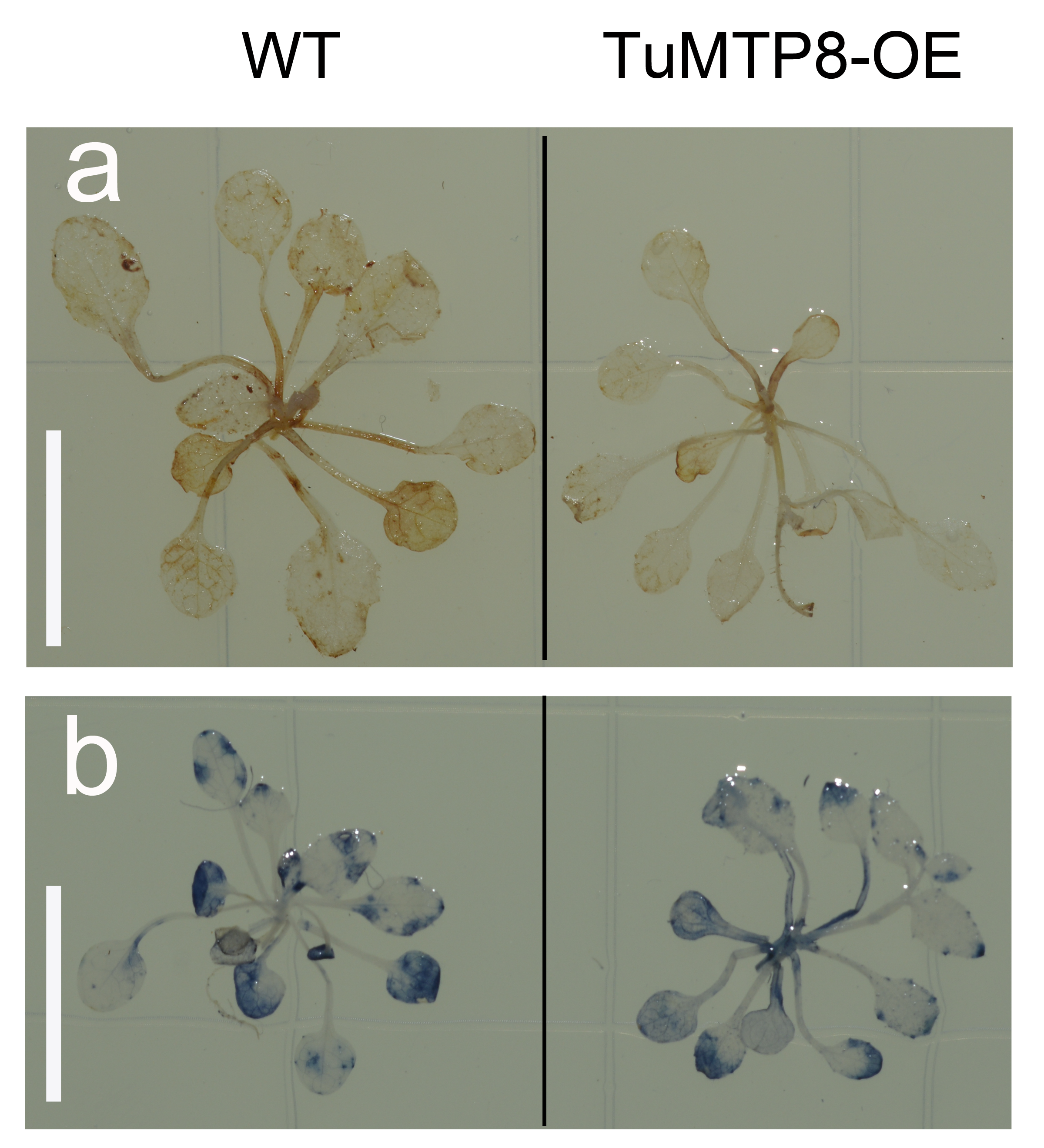

Supplement: Supplementary file 1 [file ijms-23-05683-s001.zip › Fig.S2.tif]

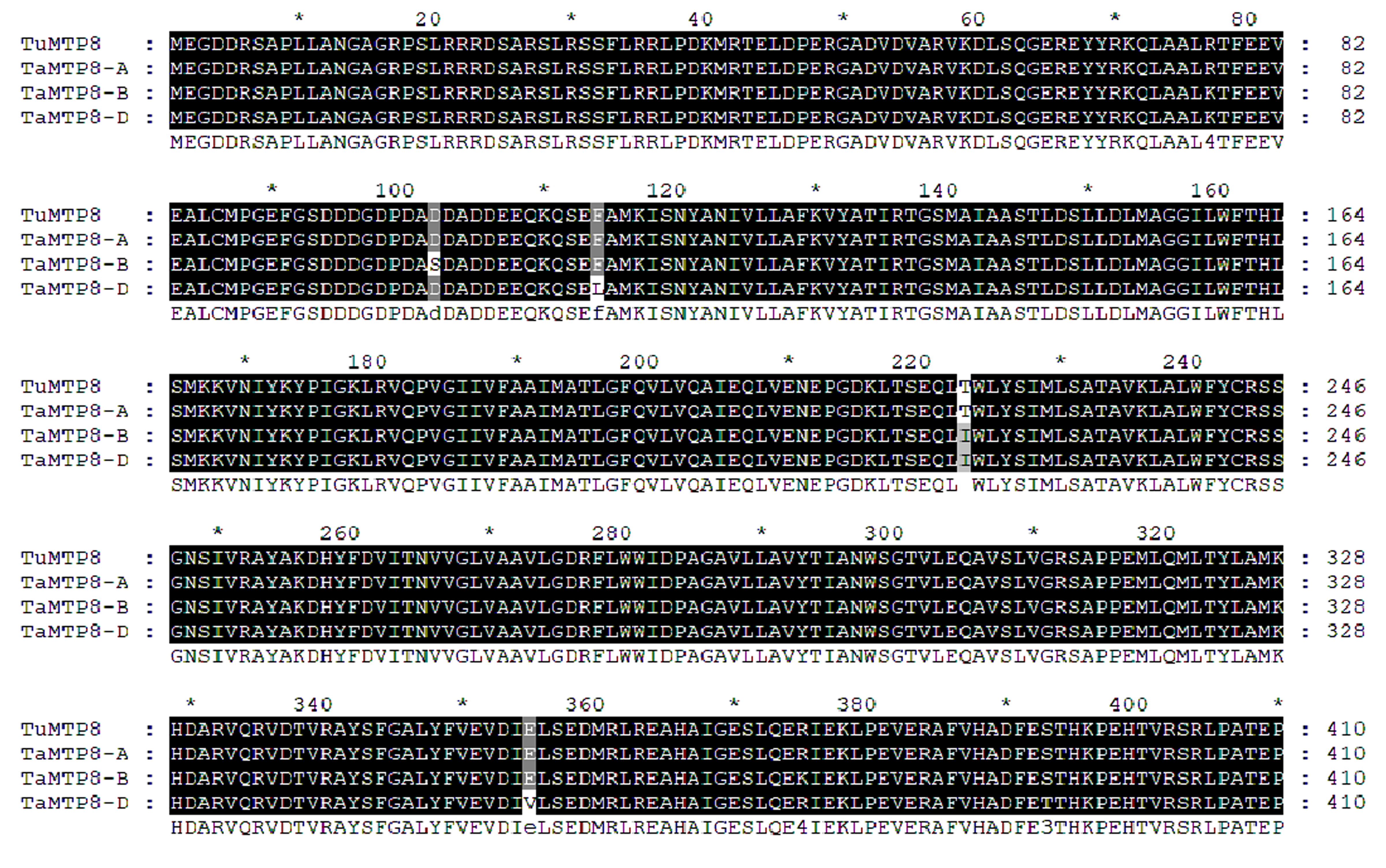

Supplement: Supplementary file 1 [file ijms-23-05683-s001.zip › Fig.S3.tif]
